# Dietary feeding of freeze-dried whole cranberry inhibits intestinal tumor development in *Apc*^min/+^ mice

**DOI:** 10.18632/oncotarget.22081

**Published:** 2017-10-26

**Authors:** Duochen Jin, Tianyu Liu, Wenxiao Dong, Yujie Zhang, Sinan Wang, Runxiang Xie, Bangmao Wang, Hailong Cao

**Affiliations:** ^1^ Department of Gastroenterology and Hepatology, General Hospital, Tianjin Medical University, Tianjin, China; ^2^ Department of Pathology, General Hospital, Tianjin Medical University, Tianjin, China

**Keywords:** cranberry, intestinal tumor, gut barrier function, epidermal growth factor receptor, Apc^min/+^ mouse

## Abstract

It is increasingly perceived that dietary components have been linked with the prevention of intestinal cancer. Cranberry is a rich source of phenolic constituents and non-digestible fermentable dietary fiber, which shows anti-proliferation effect in colorectal cancer cells. Herein, we investigated the efficacy of long-term cranberry diet on intestinal adenoma formation in *Apc*^min/+^ mice. *Apc*^min/+^ mice were fed a basal diet or a diet containing 20% (w/w) freeze-dried whole cranberry powder for 12 weeks, and the number and size of tumors were recorded after sacrifice. Our results showed that cranberry strongly prevented the growth of intestinal tumors by 33.1%. Decreased cell proliferation and increased apoptosis were observed in tumors of cranberry-fed mice. Cranberry diet reduced the expression profile of colonic inflammatory cytokines (IFN-γ, IL-1β and TNF-α) accompanied with increased levels of anti-inflammatory cytokines (IL-4 and IL-10). Moreover, the number of colonic goblet cells and MUC2 production were increased, and the intestinal barrier function was also improved. In addition, cranberry diet increased caecal short chain fatty acids concentrations, and down-regulated epidermal growth factor receptor signaling pathway. These data firstly show the efficacy and associated mechanisms of cranberry diet on intestinal tumor growth in *Apc*^min/+^ mice, suggesting its chemopreventive potential against intestinal cancer.

## INTRODUCTION

Clinical data provide an overview that colorectal cancer (CRC) has become the one of the most commonly diagnosed tumors in the overall population [1, 2]. Dietary habits with a high intake of fruits and vegetables have represented an inverse association with the risk of developing CRC, on account of the high content of fiber and phenolic compounds [3]. There have been interests in investigating the promising roles of vegetarian meals and their constituent phytochemicals against CRC both *in vitro* and *in vivo*. American cranberry (*Vaccinium* spp.) is a widely consumed berry fruit in North America due to its desirable polyphenols bioactives and berry phytonutrients. Accumulating evidence shows that daily consumption of cranberry has the potential ability to promote a healthy cardiovascular system and urinary system for its antioxidant activity [4]. Lately, there is evidence to suggest that cranberry phytochemicals have anticancer properties such as limiting prostate tumorigenesis and metastasis [5–7]. Furthermore, it has been observed that cranberry consumption in patients with prostate cancer decreased the level of serum prostate specific antigen [8] and the incidence of radiation cystitis [9]. Moreover, cranberry derived phytochemicals have been evaluated to be antineoplastic compounds in many tumor cell lines, such as in human breast cancer cells [10], lung cancer cells [11], ovarian cancer cells [12, 13], bladder cancer cells [14] and even esophageal adenocarcinoma cell lines [15, 16]. In addition, freeze-dried cranberry powder diet has been reported to alleviate inflammatory response and lipid oxidation, which is useful to individuals with the metabolic syndrome [17, 18].

Interestingly, several studies have investigated the bioavailability and metabolism of cranberry constituents in the gastrointestinal tract. Cranberry remained its antioxidant activity in the gastrointestinal tract, and 49 and 57 metabolites were detected in human plasma and urine after cranberry administration, respectively [19, 20]. The rich native A-type proanthocyanidins (PACs) of cranberry improved intestinal barrier function by stimulating goblet cells proliferation and Th2 cytokines expression [21]. Cranberry supplementation significantly attenuated colitis severity and the production of Th1 cytokines induced by dextran sodium sulfate in mice, meanwhile, gut microbiota were altered and the levels of short-chain fatty acids (SCFAs) in cecum were increased [22, 23]. Adding cranberry proanthocyanidins to elemental enteral nutrition improved and maintained luminal IgA level [24]. Furthermore, regular consumption of dietary fiber offered good protection against pathogen infection by promoting the function of the intestinal mucus barrier [25]. When it comes to complex, elusive CRC, dietary cranberry has been implicated in a decreased formation of aberrant crypt foci induced by azoxymethane in Fisher 344 male rats [26]. Cranberry extracts played a cytotoxic role in human tumor cell lines including HT-29 through apoptosis and cell cycle arrest at G1/S phase [27]. However, to date, it remains to be determined if cranberry administration inhibits spontaneous intestinal tumorigenesis *in vivo*.

*Apc*^min/+^ mice carrying heritable mutant *Apc* gene would develop multiple intestinal neoplasia that is analogous to human familial adenomatous polyposis (FAP). As a tumor suppressor gene, *Apc* gene mutations contribute to FAP and most sporadic CRC [28, 29]. This well-established animal model develops multiple polyps spontaneously in the gut, and has emerged to fulfil important roles in investigating malignant transformation in intestinal tumorigenesis [30, 31]. Disordered Apc protein leads to decreased β-catenin degradation concomitant with the activation of the Wnt pathway [32, 33]. β-catenin accumulates in the nucleus and binds to transcription factor belonging to lymphoid enhancing factor (LEF-1) family, which augments the transcriptional level of target genes including cyclin D1 gene [34]. Furthermore, *Apc* mutations are responsible for epidermal growth factor receptors (EGFR) signaling pathway activation [35]. Feng Y *et al.* highlighted that the inhibition of EGFR autophosphorylation and downstream targets (Akt kinase and extracellular signal-regulated kinase 1/2, etc.) would suppress cell proliferation and meanwhile induce cell apoptosis [36]. EGFR inhibition has already been proven to be involved in polyp growth reduction in the *Apc*^min/+^ mouse model of intestinal carcinogenesis [37]. In the present study, we investigated the inhibitory activity and underlying mechanisms of dietary cranberry against intestinal tumorigenesis in *Apc*^min/+^ mice, which might provide a translational approach to reduce the risk of CRC.

## RESULTS

### Cranberry supplementation inhibited intestinal tumor development

**Figure 1:**
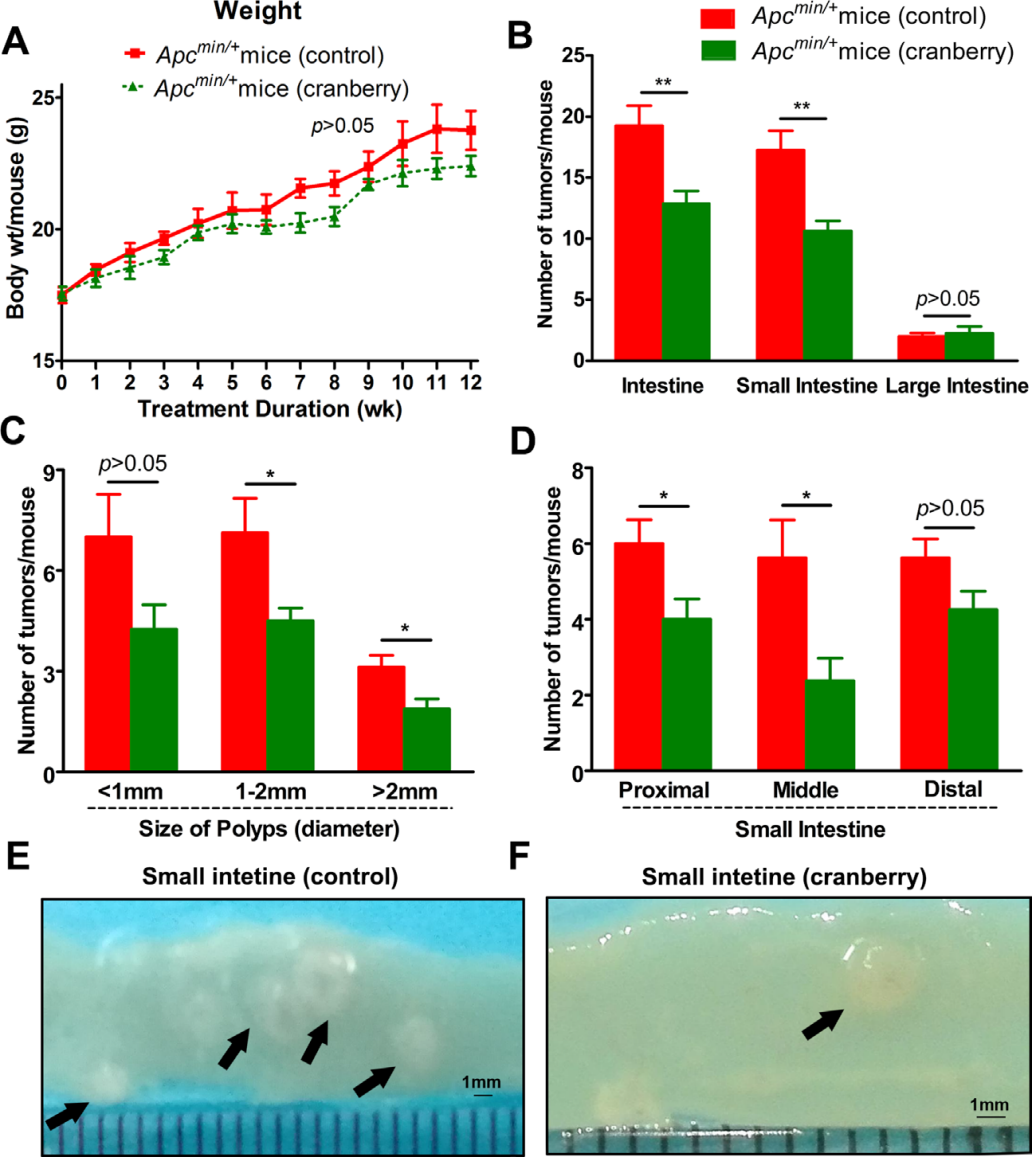
Cranberry ingestion inhibited intestinal tumor development (**A**) Dietary feeding of cranberry did not affect body weight gain in *Apc*^min/+^ mice. (**B**) The numbers of polyps per mouse in the small intestine and colon in both groups were listed. (**C–D**) Cranberry supplementation decreased the number of larger polyps (>1 mm) with the most prominent effect in proximal and middle sections of small intestine. Columns represented as mean from the ten mice in each group, bars represented as standard deviation. ^**^p < 0.01 and ^*^p < 0.05, cranberry diet-fed *vs* basal diet-fed *Apc*^min/+^ mice. (**E**–**F**) The representative gross appearance of intestinal tumors from both groups was shown after 12-week experiment.

All mice were regularly monitored to investigate the body weight and consumption of food and water during the experiment. No significant difference was found in food consumption or body weight between the control and cranberry groups throughout the study (Figure 1A). There was no mortality throughout the treatment period in any group. Furthermore, no macroscopic alterations indicative of toxicity were observed in any organs of cranberry-treated mice, including the liver, kidney and lung.

The control mice developed an average of 19.25 intestinal tumors per mouse, which were mostly populated in the small intestine. Dietary feeding of cranberry significantly decreased the total number of intestinal polyps by 33.1% (19.25 ± 4.62 *vs* 12.88 ± 2.90, *p* < 0.01; Supplementary Table 1, Figure 1B). More specifically, the prominent effect of cranberry on the decrease in larger polyps (>1 mm) of small intestine was observed in size distribution analysis (<1 mm, *p* > 0.05; 1–2 mm, *p* < 0.05; >2 mm, *p* < 0.05; Figure 1C). Further, proximal and middle portions of small intestine showed 33.3% (*p* < 0.05) and 57.7% (*p* < 0.05) reduction in the numbers of polyps by cranberry, respectively (Figure 1D). However, there were no significant differences for the numbers of distal and colonic tumors between the two groups (Figure 1B and 1D). Both groups could develop adenomas with or without low-grade dysplasia at all segments of the intestine, which had no significant difference with respect to tumor stage. These data suggested the inhibitory effect of cranberry on tumor growth without any toxicity in *Apc*^min/+^ mice.

### Cranberry diet inhibited cell proliferation and induced apoptosis in intestinal tumors

**Figure 2:**
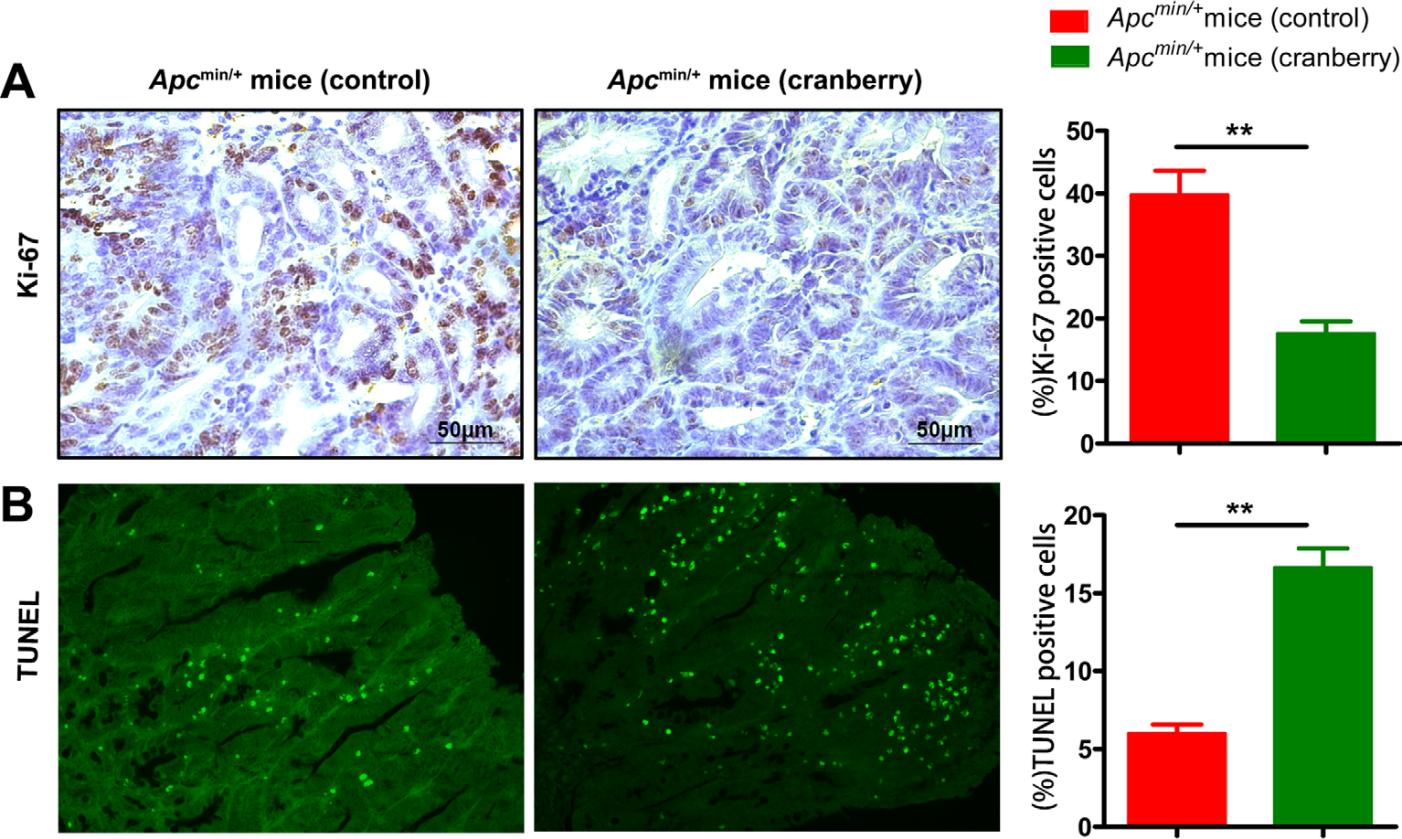
Cranberry supplementation inhibited proliferation and promoted apoptosis in intestinal tumors (**A**–**B**) Middle small intestinal sections from basal diet-fed and cranberry diet-fed mice were stained with Ki-67 and TUNEL, wherein brown-stained cells demonstrated Ki-67 immunostaining (400× magnifications), green staining represented apoptotic cells (200× magnifications). Scale bars, 50 μm. Both assays were quantified by counting percent positive rate of tumor cells’ nuclei at 5 randomly selected fields from each section. Values are means from ten mice in each group with their standard errors. ^**^p < 0.01, cranberry diet-fed *vs* basal diet-fed *Apc*^min/+^ mice. *n* = 10/group.

Cell proliferation and apoptosis have been implicated in evaluating intestinal tumor development in *Apc*^min/+^ mice. The level of proliferation was reflected by Ki-67 immunostaining of sections from middle small intestine Swiss rolls, which is used as a prognostic marker in human neoplasia. As shown in Figure 2A, intestinal tissue sections of the cranberry diet-fed *Apc*^min/+^ mice exhibited a significant decrease in the number of proliferative cells within tumors, compared with those of the basal diet-fed mice (109.67 ± 10.78 *vs* 55.33 ± 7.41, *p* < 0.01). The level of apoptosis in adenomas of *Apc*^min/+^ mice was determined by TUNEL, and cranberry group had more apoptotic cells in tumors compared with mice on control diet (6.33 ± 0.47 *vs* 16.67 ± 1.70, *p* < 0.01, Figure 2B).

### The expression profile of colonic inflammation mediators was regulated by cranberry

**Figure 3:**
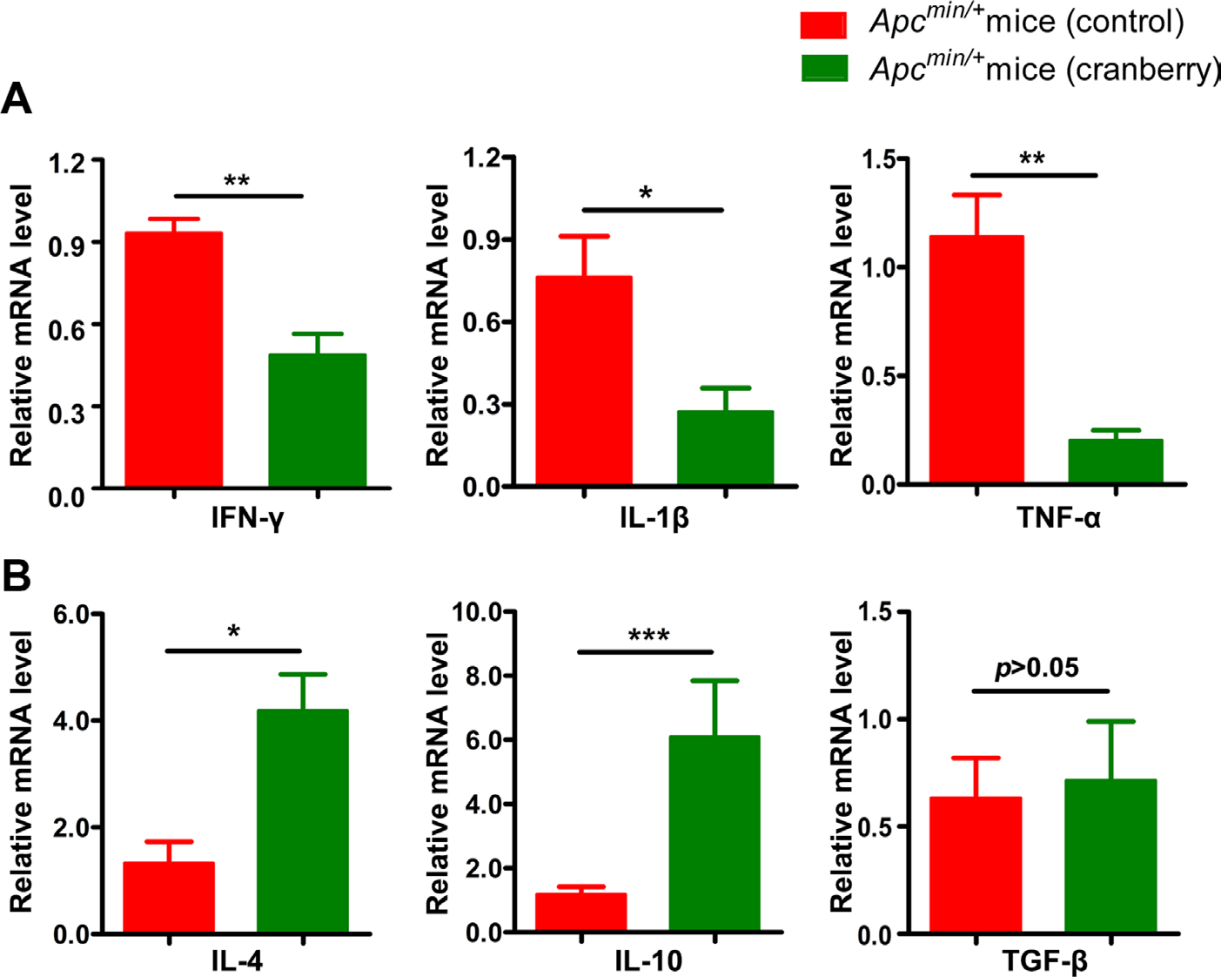
Dietary cranberry relieved chronic inflammation in colon (**A**–**B**) RNA was extracted from tumor-adjacent tissue in colon for real-time quantitative PCR analysis. The relative mRNA expression of inflammatory cytokines including IFN-γ, IL-1β, TNF-α, IL-4, IL-10 and TGF-β was presented. Data was expressed as fold change compared with the control group (100%). ^*^p < 0.05, ^**^p < 0.01, ^***^p < 0.001, cranberry diet-fed *vs* basal diet-fed *Apc*^min/+^ mice. *n* = 10/group.

Chronic inflammation of the intestinal mucosa has been suggested to play a crucial role in regulating immune response to initiate or promote CRC development [38]. In this experiment, we investigated the effect of cranberry supplementation on cytokine profiles in the pericarcinous tissues in colon. We observed that the mRNA expression levels of IFN-γ, IL-1β and TNF-α mRNA were significantly down-regulated, whereas IL-4 and IL-10 were remarkably increased in the colonic mucosa from cranberry diet-fed *Apc*^min/+^ mice compared with control without obviously affecting TGF-β (Figure 3A and 3B). These data suggested that the inhibition of pro-inflammatory cytokines expression and the promotion of anti-inflammatory cytokines by cranberry diet might play a role in the process of inflammation during intestinal tumorigenesis in *Apc*^min/+^ mice.

### Cranberry feeding improved intestinal mucosal barrier function in *Apc*^min/+^ mice

**Figure 4:**
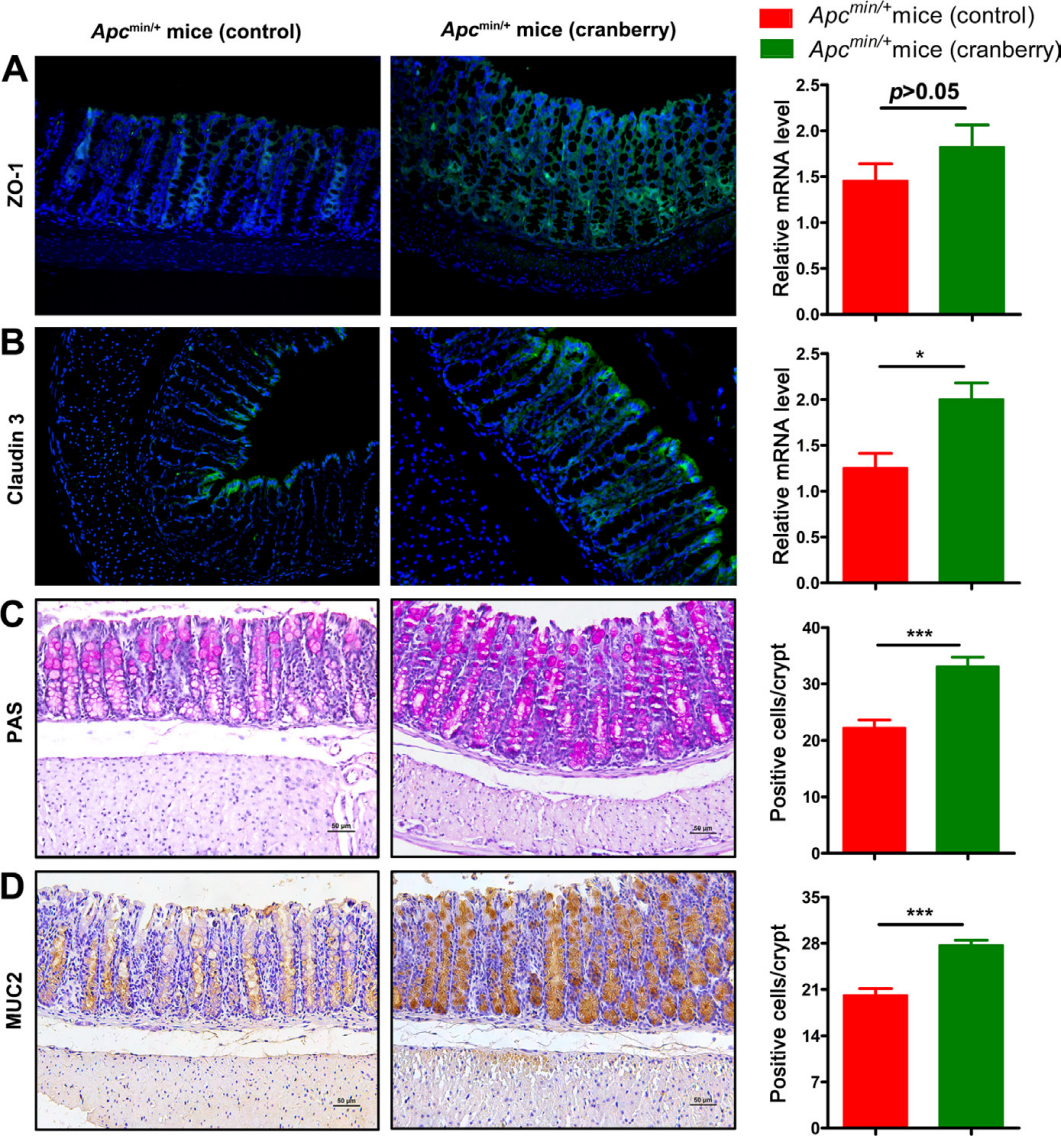
Cranberry supplementation protected colonic barrier function (**A**) Paraffin-embedded colon tissues were used to determine ZO-1 distribution by immunofluorescence stain using an anti-ZO-1 antibody and FITC-labeled secondary antibody and visualized using fluorescence microcopy (green staining; 200×). Nuclei were stained with DAPI (blue staining). Real-time PCR analysis of ZO-1 expression in the cell membranes of colonic epithelial cells was shown. (**B**) Claudin 3 distribution was showed analogously by immunofluorescence stain and real-time PCR. (**C**–**D**) PAS and MUC2 in the colon tissues of both groups were assessed using immunostaining (200×). Scale bars, 50 μm. The numbers of positively stained cells were determined by counting the absolute number of positive stained cells in at least 300 colonic crypts for each mouse. ^***^p < 0.001, ^*^p < 0.05, cranberry diet-fed *vs* basal diet-fed *Apc*^min/+^ mice. *n* = 10/group.

Previous studies have suggested that colitis mouse models and patients with ulcerative colitis present a reduced number of goblet cells and thus a thinner mucus layer [39, 40]. In the present study, immunostaining and RT-PCR were used to assess the distribution of tight junction protein, zona occludens 1 (ZO-1) and claudin 3 in the middle small intestine and the colon, respectively (Figure 4A and 4B; Supplementary Figure 1) [41]. Control group was associated with a leaky tight junction, meanwhile, cranberry supplementation restored impaired epithelial tight junction [42]. Cranberry-fed group showed higher gene expression levels of ZO-1 and claudin 3 than control group in the small intestine (*p* < 0.01, *p* < 0.05; Figure 4A and 4B). PAS staining showed that diet supplementation with cranberry increased the number of goblet cells compared with control diet (22.35 ± 4.46 *vs* 33.11 ± 4.58, *p* < 0.001, Figure 4C). As a major component of inner mucus layer, MUC2 mucin produced by goblet cells was up-regulated by cranberry feeding (16.13 ± 2.71 *vs* 27.71 ± 1.83, *p* < 0.001, Figure 4D). Cranberry-treated mice were associated with longer villi and deeper crypts compared with control mice (Figure 4 and Supplementary Figure 1). The results suggested that cranberry might be effective in influencing the number of goblet cells and gut mucin production, and ultimately enhancing the integrity of the gut barrier.

### Cranberry feeding increased caecal concentrations of SCFAs in *Apc*^min/+^ mice

Fibre containing diet influences the production and absorption of SCFAs, particularly propionic and butyric acids. SCFAs, which are formed by microbial degradation of dietary fibres in colon, have capacity to reduce low-grade inflammation [43, 44]. In this experiment, we found that the addition of cranberry diet to *Apc*^min/+^ mice made no significant difference in the weight of caecal content (*p* > 0.05, Table 1). The concentration of acetic acid was highest, followed by propionic and butyric acids. These caecal concentrations of the three SCFAs were 2.9-3. 2-fold higher than that of the control group (*p* < 0.05). The observed results highlighted that SCFAs had been postulated to elucidate the underlying link between fibre containing diet and prevention of CRC.

**Table 1:** Effects of cranberry diet on the weight of caecal content and SCFAs concentrations

	*Apc*^min/+^ mice (control)		*Apc*^min/+^ mice (cranbery)	
	Mean	SEM	Mean	SEM
Caecal content weight (g)	0.15	0.07	0.06	0.05
Acetate (μM/g WW)	7.36	1.68	23.74	8.74^*^
Propionate (μM/g WW)	5.45	1.14	15.92	5.54^*^
Butyrate (μM/g WW)	3.71	0.78	11.47	4.05^*^

WW, wet weight. SCFAs, short-chain fatty acids. Values represented the means with their standard errors, *n* = 10/per group. ^*^p < 0.05.

### Cranberry feeding down-regulated EGFR signaling in *Apc*^min/+^ mice

**Figure 5:**
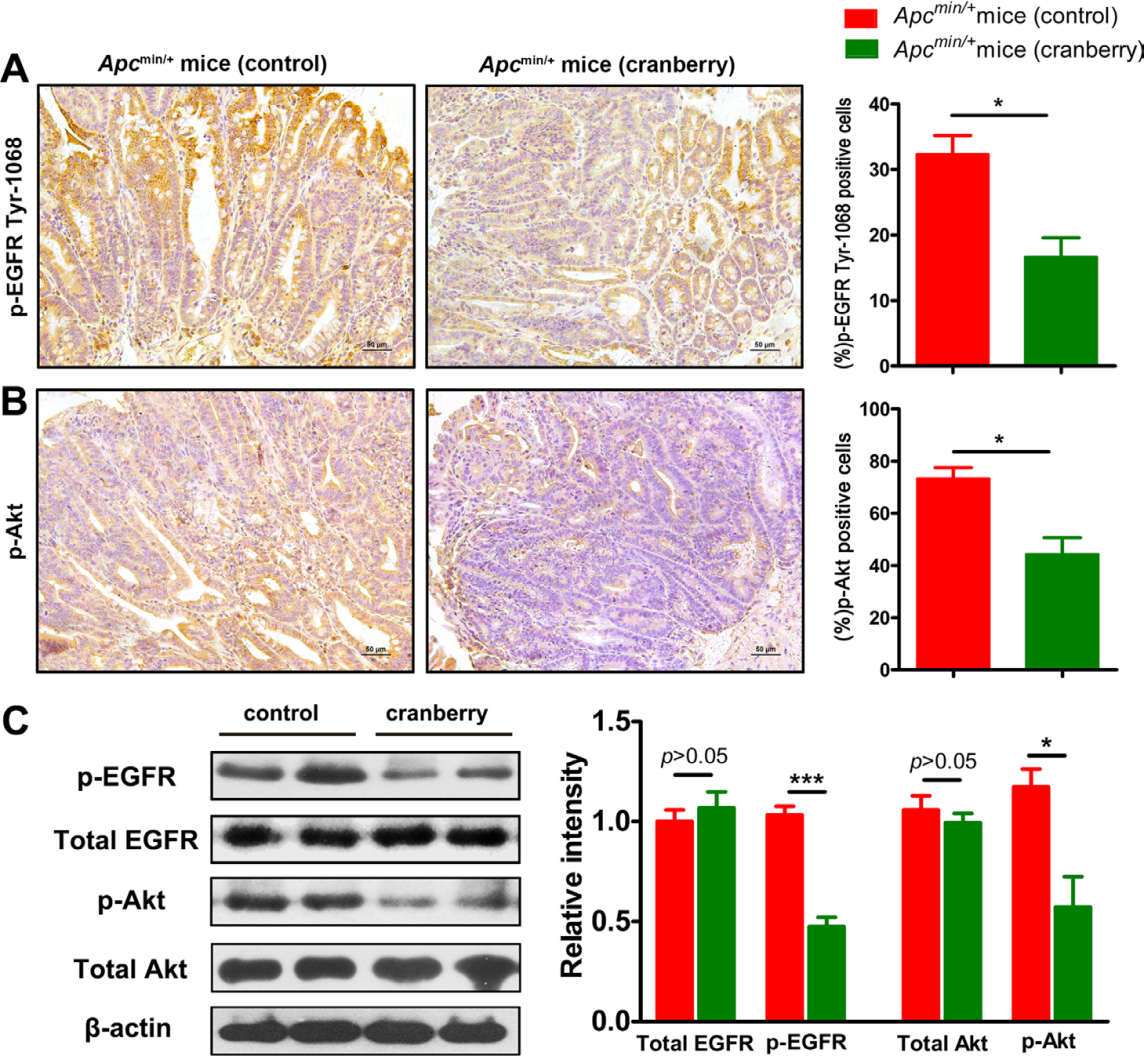
Cranberry supplementation down-regulated EGFR signaling pathway in intestinal tumors (**A**–**B**) p-EGFR and p-Akt from the diatal small intestine of both groups were shown by immunohistochemical staining (400×). Scale bars, 50 μm. Data were semiquantified as mean percentage of positive cells at five randomly selected fields. (**C**) Protein lysates from tumors were analyzed by Western blot analysis using anti-total EGFR and p-EGFR antibodies, and anti-total Akt and p-Akt antibodies, which anti-β-actin antibody was used as an internal control for total protein. The fold changes were calculated by comparing the relative densities of total EGFR, p-EGFR and p-Akt bands to corresponding β-actin bands from the same mouse. Proteins were quantified by densitometry using Image J to calculate the average ratio, and the average ratio in control was set as 100%. Columns, means from at least six mice in each group; bars, standard deviation. ^*^p < 0.05, ^***^p < 0.001, cranberry diet-fed *vs* basal diet-fed *Apc*^min/+^ mice. *n* = 8/group.

The effects of cranberry feeding on EGFR signaling pathway involved in tumor development were further investigated. Immunostaining supported that phosphorylation of EGFR and Akt in intestinal tumors from distal small intestine were suppressed by cranberry supplementation. It was observed that the average percentages of p-EGFR-positive cells in cranberry diet-fed and basal diet-fed groups were 16.67 ± 4.11 *vs* 32.33 ± 4.03 (*p* < 0.05, Figure 5A) and p-Akt stained cells were 44.33 ± 8.99 *vs* 73.33 ± 5.91 (*p* < 0.05, Figure 5B). However, immunostaining showed that there was no difference in the activation of Wnt/β-catenin signaling between the two groups (*p* > 0.05, Supplementary Figure 2).

Western blot analyses were consistent with immunostaining results. We compared the relative density of the protein band for total EGFR, p-EGFR, total Akt, and p-Akt to those of internal control bands from the same mouse's tumor tissue lysate. The fold changes of the average protein band ratio in cranberry diet-fed group were calculated since the control group’ was set as 100%. The levels of phosphorylated EGFR and Akt were reduced in tumors of cranberry diet-fed group when compared to controls (*p* < 0.001; *p* < 0.05, Figure 5C). No differences were found in the total amounts of EGFR and Akt. All these results suggested that cranberry supplementation suppressed the activation of EGFR signaling in *Apc*^min/+^ mice.

## DISCUSSION

It is increasingly perceived that dietary components have been linked with the prevention of CRC [25, 45]. Among them, fruits and vegetables have emerged to fulfil important roles in improving health. Berries promisingly have raised interests for their high phytochemical and fibre content [46–48]. The present study showed the inhibitory effects of feeding cranberry supplemented diet on the intestinal polyposis in the *Apc*^min/+^ mouse model, which justified further research about how berry phytonutrients prevented CRC *in vivo*. It was highlighted that dietary cranberry would beneficially modulate colonic inflammation and caecal SCFAs, inhibit activation of EGFR signaling and regulate proliferation and apoptosis of tumour cells possibly due to the fermentable fibre components and phenolic compounds in berry.

Recent years researchers have raised interests in botanical dietary therapeutics, corresponding with an association that plants, fruits and vegetables contain intricate components with multifunctional roles, which may better prevent or postpone elusive chronic diseases than single active pharmaceutical ingredients. As chemopreventive dietary constituent, cranberry fruit (*Vaccinium macrocarpon* Ait.) is a promising dietary source of phytochemicals, because of richness in flavonoids such as anthocyanins glycosides, flavonol glycosides, and proanthocyanidin (PAC) oligomers; organic and phenolic acids such as benzoic acid and ursolic acid, etc. [49–51]. In particular, proanthocyanidinsin cranberries contain “A-type” bonds compared with the B-type PACs present in most other fruits [46]. Polyphenols from cranberry have emerged to fulfil putative roles in chronic diseases, i.e. type 2 diabetes, heart disease and various cancers for their high antioxidant capacities [52]. Cranberry “A-type” PAC could better contribute to pathogenic *Escherichia coli* agglutination and then decrease the invasion of enterocytes [53]. Most of procyanidins are degraded into multiple phenolic compounds as biologically active forms under the action of gut microbiota. Furthermore, dietary fiber of cranberry may influence the gut microbiota, which is essential to intestinal barrier function [25]. Cranberry bioactives are possible contributors to limit carcinogenesis in a complementary fashion. In this study, the consumption patterns of CRC patients are imitated appropriately, because 20% dietary supplementation level is achievable for human being [54–56], which offers a fine prospect in achievement transform. In addition, effects require further elaboration to determine the dose-response relationships of lower levels of cranberry supplementation to health benefits.

Chronic inflammation and excessive immune signaling receive attention for the causes of dysregulation of cell proliferation in *Apc*^min/+^ mice, which accompanied with the up-regulation of inflammation genes [57]. The levels of intestinal inflammation cytokines are used to assess the development and progression of inflammatory responses in CRC patients [58, 59]. The present study found that inflammatory mediators of the colon were regulated by dietary cranberry in *Apc*^min/+^ mice, which exhibited a reduced intestinal inflammatory response. These mice fed by cranberry had a lower level of inflammation. Phytochemicals including fibre and phenolic compounds in cranberry are known to influence inflammatory responses by way of exerting a myriad of cellular effects [60–62]. These effects include release of antioxidant potential and induction of an anti-inflammatory effect by modulating NF-κB signaling [63, 64]. Therefore, the combined effects of phytochemicals in cranberry diet beneficially attenuated intestinal immune response in *Apc*^min/+^ mice. Gut barrier separates the host from luminal microorganisms and noxious molecules while absorbing nutrients. Mucus layer, mainly comprising MUC2, is the front line of innate immune defense. Gut barrier properties may be destroyed due to unbalancing pro- and anti-inflammatory mediators in the mucosa [42, 65, 66]. In this study, cranberry ingestion stimulated goblet cells to produce MUC2 and promoted epithelial intercellular junctions to maintain gut barrier.

Substantial evidence indicates that a Western-style diet low in dietary fiber is associated with a high risk of colorectal carcinogenesis [67]. Following the consumption of cranberry fibre, the anti-cancer potential may have been driven by microbial metabolic products, such as SCFAs [68]. SCFAs are the end productions of non-digestible dietary carbohydrates under the fermentation of intestinal microflora [22]. SCFAs, particularly butyrate, have been regarded as chemopreventive agents which play plurifunctional roles in the colonocytes. The current working hypothesis is that butyrate provides energy for epithelial cells [69, 70] and enhances gut barrier integrity [71]. Furthermore, butyrate exerts anti-inflammatory effects within epithelium through the modulation of inflammatory signaling pathways including the inhibition of histone deacetylase activity. All these actions play a comprehensive role in influencing the dynamic balance of gut microbiota towards commensal bacteria such as butyrate-producing bacteria [70, 72, 73]. In the present study, as a consequence of increased microbial activity, caecal SCFA concentrations (acetate, propionate and butyrate) were increased in cranberry-supplemented group compared to the control. And, we did not find that proanthocyanidin- rich dietary fiber could increase the caecal content weight similar to those in other studies [74, 75]. Furthermore, butyrate has been implicated in the decreased expression of pro-inflammatory mediators, as well as increased expression of anti-inflammatory mediators, which was observed by Jakobsdottir G *et al*. [74]. Butyrate and its role in CRC prevention need to be excavated for its clinical use any further.

Anthocyanins, proanthocyanidins, and flavonol glycosides have demonstrated enhanced antiproliferative interactions synergistically or additively [16, 76]. In the present study, daily consumption of cranberry for 12 weeks reduced proliferation and induced apoptosis in colorectal tissue of *Apc*^min/+^ mice. The activation of EGFR is essential for accelerating cell cycle progression, promoting cell proliferation and impeding cell apoptosis by phosphorylating (activation) its downstream targets (PI3K/AKT and Ras-ERK) in many tissue types [77]. Activating EGFR/AKT signaling is known to promote tumorigenesis, and EGFR signalinghas regarded as a promising target to control tumor development [16]. In this study, the dietetic treatment with cranberry was found to beneficially regulate cell proliferation and apoptosis, and meanwhile impede the phosphorylation of EGFR and its downstream Akt in intestinal tumors in *Apc*^min/+^ mice, compared with basal diet. However, it remains to be determined the mechanisms of the inhibitory effects of EGFR pathway by cranberry nutrients.

Overall, this study suggested that dietary administration of cranberry inhibited intestinal tumor development in the *Apc*^min/+^ mouse model. Cranberry exerted its effects probably by decreasing inflammatory cytokine production and promoting intestinal barrier function while simultaneously regulating EGFR signaling, associated with intestinal proliferation and apoptosis for its fiber-derived SCFAs. In summary, an appropriate dietary intervention, as a new treatment strategy, in combination with pharmaceuticals, may have benefit on preventing intestinal cancer in high-risk populations.

## MATERIALS AND METHODS

### Animals and diets

Female *Apc*^min/+^ mice aged 4 weeks on C57BL/6J background (*n* = 20) were obtained from the Animal Model Institution of Nanjing University, P. R. China. The genotypes of the mice were screened using PCR methods to identify Min genotype [78]. Animals were housed in humidity- and temperature-controlled plastic cages with 12/12 h light/dark cycle (temperature: 25°C; humidity: 50%; lights off at 18:00) under specific pathogen free (SPF) circumstances. The animals were randomly divided into two groups (10 mice per group, 5 mice per cage). Control group was fed AIN-93G control diet continuously while the cranberry-treated group was fed 20% cranberry powder (wt/wt) mixed in AIN-93G diet *ad libitum* for 12 weeks by reference to dosages in published reports [22, 48, 56]. Freeze-dried whole cranberry powder was purchased from commercial vendor Peak Season Foods (Nampa, Idaho, USA), and its nutrition facts were showed in Table 2. The rodents were weighed once a week and monitored daily for any signs of toxicity throughout the 12 week treatment period. The animals were starved overnight and then killed by CO_2_ inhalation at the end of 12 weeks. All the animals were starved overnight before sacrifice in order to clean the bowel and obtain intestinal tissues more easily. Caecal contents were snap-frozen and stored in refrigerators at −80°C until analysis for SCFA content. All experimental procedures were performed under the guidelines of the Institutional Animal Care and Use Committee at Tianjin Medical University, Tianjin, P. R. China.

**Table 2:** Nutrition facts of freeze-dried whole cranberry powder

Serving Size 10 Grams	
Serving Per Container 23	
Amount Per Serving	
Calories 38	Calories from Fat 0
	% Daily Values^*^
Total Fat 0 g	0%
Saturated Fat 0 g	0%
Trans Fat 0 g	
Cholesterol 0 mg	0%
Sodium 2 mg	0%
Total Carbohydrate 9 g	3%
Dietary Fiber 2 g	8%
Sugars 5 g	
Protein 0 g	0%
Vitamin A 1%	Vitamin C 3%
Calcium 0%	Iron 0%

^*^Percent Daily Values are based on a 2,000 calorie diet.

### Measurement of tumors and tissue collection

The intestine was immediately excised from each mouse after sacrifice as previously described [79]. The intestines of all mice were opened longitudinally and then rinsed well with sterile ice-cold PBS solution to remove the intestinal contents. The small intestine was divided into three equal sections, i.e., proximal, middle, and distal, and the colon. Afterwards, all tissue sections of the small intestine and colon were viewed and recorded by the same veterinary pathologist blinded to the experiment with a steel rule under an Olympus SZX7 stereo dissecting microscope. The total numbers of polyps in each section were counted. The size of each polyp was measured and categorized as small (<1 mm), medium (1–2 mm), or large (>2 mm). Swiss-rolled middle small intestine and colon as well as hepatic and kidney tissues were fixed in 10% neutral-buffered formalin until further analysis for preparing Paraffin-embedded tissue sections. Paraffin-embedded intestinal Swiss rolls containing tumors were stained with hematoxylin and eosin (H&E) for tumor stage or used for immunohistochemical staining. Adenomas from middle small intestinal tissues were excised and snap-frozen rapidly, and stored at −80°C for later analysis of protein expression.

### Periodic acid Schiff (PAS) staining

1% Periodic acid solution (Sigma-Aldrich) was used to incubate deparaffinised colonic sections for 10 min. And then Schiff reagent (Sigma-Aldrich) was used for incubation for 40 min. The PAS-stained sections were counterstained with Hematoxylin for 2–5 min. Extensive PBS solution was used to rinse well between each step.

### Histopathology and immunohistochemistry

Formalin-fixed, paraffin-embedded intestinal tissue blocks were cut into 4-μm slices by a microtome for immunostaining. Thereafter, intestinal tissue slices were deparaffinised in xylene and rehydrated in graded ethanol. Antigens were retrieved in Antigen Unmasking Solution (Vector laboratories, Inc. Burlingame, CA, USA) for 15 min. Endogenous peroxidase activity was quenched by immersing in 3% hydrogen peroxide for 10 min. 5% goat serum was used to block non-specific binding in Tris-buffered saline for 1 h at room temperature. The tissue sections were incubated with primary antibodies, rabbit anti-Ki-67 (ab16667, Abcam, Cambridge, MA, USA), phospho-EGFR (Tyr1068) (CST3777, Cell Signaling technology, Boston, MA, USA), phospho-Akt (p-Akt, Ser473) (CST4060, Cell Signaling technology, Boston, MA, USA), β-catenin (Santa Cruz Biotechnology, Inc., Santa Cruz, CA, USA), cyclin D1(ab134175, Abcam, Cambridge, MA, USA) and rabbit anti-MUC2 (Santa Cruz Biotechnology, Inc), overnight at 4°C in a humidity-controlled chamber. Washed sections were then incubated with appropriate horseradish peroxidase (HRP)-labeled second antibodies for 30 min followed by incubation with 3, 3′-diaminobenzidine for color development. The sections were viewed blindly (400× or 200× magnification) under the light microscope by the same pathologist (YJZ). At least five fields searched for each tumor without any overlap were viewed to count the numbers of positive cells (brown staining). All tumors in each section were analyzed to calculate the final numbers of positive cells. Hence, the proliferation index was determined as the number of Ki-67-positive cells × 100 / total number of cells.

### TUNEL assay

Terminal deoxynucleotidyl transferase dUTP nick end labeling (TUNEL) assay was performed to detect apoptosis in tumors of middle small intestine. Paraffin-embedded sections containing tumors were deparaffinized, and stained for apoptotic nuclei using an *In Situ* Cell Death Detection Kit (Roche Diagnostics) based on manufacturer's protocols. To quantitate the average number of apoptotic cells in each group, five fields from each section were randomly chosen with 200× magnification (*n* = 10/ group).

### Real-time quantitative PCR analysis

Total RNA was extracted from tumor-adjacent tissues in distal small intestine and colonusing the RNeasy mini kit (Qiagen, Carlsbad, CA, USA) and reverse-transcribed using the TIANScript Reverse Transcription Kit (TIANGEN, Inc. Beijing, China). Real-time PCR was performed to measure the levels of cytokines, ZO-1 and claudin 3 using Taqman Gene Expression Master Mix. The Oligonucleotide primer sequences were showed in Table 3. Glyceraldehyde-3-phosphate dehydrogenase (GAPDH) was used as a house-keeping gene to normalize the relative abundance of targeted genes at the level of mRNAs. Real-time polymerase chain reaction (PCR) was carried out for amplification on a StepOne Plus real time PCR instrument (Applied Biosystems, Carlsbad, CA) following the manufacturer's recommendations with all cDNA products analyzed in triplicate. Gene expression of each transcript was analyzed using the standard ∆∆CT method to calculate fold-changes which were normalized to the housekeeping genes for each sample.

**Table 3:** Gene sequences of primers in the present study

Primers	Sequence
GAPDH	Forward 5′-TGTGTCCGTCGTGGATCTGA-3′
	Reverse 5′-CCTGCTTCACCACCTTCTTGA-3′
IFN-γ	Forward 5′-GCATCTTGGCTTTGCAGCT-3′
	Reverse 5′-CCTTTTTCGCCTTGCTGTTG-3′
IL-1β	Forward 5′-GTGGCTGTGGAGAAGCTGTG-3′
	Reverse 5′-GAAGGTCCACGGGAAAGACAC-3′
TNF-α	Forward 5′-ACTCCAGGCGGTGCCTATG-3′
	Reverse 5′-GAGCGTGGTGGCCCCT-3′
IL-4	Forward 5′-CGAATGTACCAGGAGCCATATC-3′
	Reverse 5′-TCTCTGTGGTGTTCTTCGTTG-3′
IL-10	Forward 5′-TGGACAACATACTGCTAACCG-3′
	Reverse 5′-GGATCATTTCCGATAAGGCT-3′
TGF-β	Forward 5′-GCTGAACCAAGGAGACGGAAT-3′
	Reverse 5′-GCTGATCCCGTTGATTTCCA-3′
ZO-1	Forward 5′-GGGCCATCTCAACTCCTGTA-3′
	Reverse 5′-AGAAGGGCTGACGGGTAAAT-3′
claudin 3	Forward 5′-CCTGTGGATGAACTGCGTG-3′
	Reverse 5′-GTAGTCCTTGCGGTCGTAG-3′

### Western blot analysis

Tumors from distal small intestinal tissues were excised and stored in refrigerators at −80°C. The total cellular lysates of these adenomas were prepared by sonication and RIPA buffer, during which 10 μL/mL of proteinase inhibitor cocktail and phosphatase inhibitor cocktail (Sigma, St. Louis, MO) were added respectively. After homogenization and centrifugation (12,000 g, 4°C, 15 min), protein concentration of the resulting lysate was determined by Bicinchoninic acid protein assay (Thermo Scientific Inc.). Proteins were separated by SDS-polyacrylamide gel electrophoresis and then transferred onto a PVDF membrane. Primary antibodies, including rabbit polyclonal antibodies against EGFR, phospho-EGFR, Akt, and phospho-Akt, were used to perform western blot, and later blotted with secondary antibodies (anti-rabbit IgG peroxidase conjugates). Anti-β-actin antibody was employed to assess total protein loading of cellular lysate. The chemiluminescent signal of the PVDF membrane was detected by X-ray films with ECL (GE Healthcare, Bucks, UK) and analyzed with an image processor program (Image J), which contributed to determine the intensity of the targeted band and internal control band in each individual mouse.

### Caecal SCFAs detection using gas chromatography

The SCFAs including acetic, propionic and butyric acids in the caecal contents of mice were analyzed using gas chromatography (GC) as previously described with slight modification [56, 74]. Briefly, SCFAs were extracted from frozen caecal contents and injected into the GC system (Agilent 7890A) equipped with a HP-INNOWAX capillary column (30 m × 0.25 mm × 0.25 μm; Agilent Technologies, USA) for chromatographic separation. Samples (1 uL each) were injected into the capillary column by split-injection mode with a split ratio of 1:1. Nitrogen was used as carrier gas at a constant flow of 1 mL/min. Injector temperature was maintained at 220°C. After an initial temperature of 70°C for 2 min, the oven temperature was increased to 150°C at a rate of 10°C/min, and then increased by 15°C/min and finally kept at 230°C for 5 min. Different peaks were identified according to their respective retention times using acetic, propionic and butyric acids GC standards (Sigma-Aldrich, USA). Agilent Chemstation software was used for data collection.

### Statistical analysis

All continuous variables were presented as mean ± SEM. Statistical comparisons of the multiplicity of intestinal tumors were analyzed by two-tailed Student's *t*-test with the GraphPad Prism version 5.00 (GraphPad Software, Inc.). The percentage of positively stained cells and the fold changes of the ratio for the relative density of bands in western blot analysis were performed using Student's *t*-test. Student's *t*-test was also used to determine differences in body weight between groups. Differences were considered statistically significant with *p* < 0.05.

## SUPPLEMENTARY MATERIALS FIGURES AND TABLE



## Abbreviations

Apc: adenomatous polyposis coli gene
CRC: colorectal cancer
EGFR: epidermal growth factor receptors
FAP: familial adenomatous polyposis
GAPDH: glyceraldehyde-3-phosphate dehydrogenase
GC: gas chromatography
HRP: horseradish peroxidase
IL: interleukin
IFN: interferon
Min: multiple intestinal neoplasia
MUC: mucin
PACs: A-type proanthocyanidins
PAS: Periodic acid-schiff
PBS: Phosphate buffer saline
PCR: real-time polymerase chain reaction
SCFA: short chain fatty acid
SPF: specific pathogen free
TNF: tumor necrosis factor
TUNEL: terminal deoxynucleotidyl transferase dUTP nick end labeling assay
ZO-1: zona occludens 1
