# Supplementary material for: Dietary feeding of freeze-dried whole cranberry inhibits intestinal tumor development in Apcmin/+ mice
Source: Oncotarget. 2017 Oct 26;8(58):97787–800. doi: 10.18632/oncotarget.22081 (PMC5716691; doi:10.18632/oncotarget.22081)
Supplement: Supplementary file 1 [file oncotarget-08-97787-s001.pdf]

Dietary feeding of freeze-dried whole cranberry inhibits intestinal tumor development in *Apc*$^{min/+}$ mice

SUPPLEMENTARY MATERIALS

Supplementary Table 1: Effects of cranberry on intestinal tumor multiplicity in *Apc*$^{min/+}$ mice ($\bar{X} \pm S$, $n = 10$)

	Apc$^{min/+}$ mice (control)	*Apc*$^{min/+}$ mice (cranberry)
**Total number**	19.25 ± 4.62	12.88 ± 2.90**
**Small intestinal**	17.25 ± 4.50	10.63 ± 2.39**
<1 mm	7.00 ± 3.59	4.25 ± 2.05
1–2 mm	7.13 ± 2.90	4.5 ± 1.07*
>2 mm	3.13 ± 0.99	1.88 ± 0.83*
Proximal	6.00 ± 1.77	4.00 ± 1.51*
Middle	5.63 ± 2.83	2.38 ± 1.89*
Distal	5.63 ± 1.41	4.25 ± 1.39
**Colon**	2.00 ± 0.76	2.25 ± 1.58

$^{**}p < 0.01$ and $^{*}p < 0.05$, cranberry-fed *vs* control *Apc*$^{min/+}$ mice, respectively.

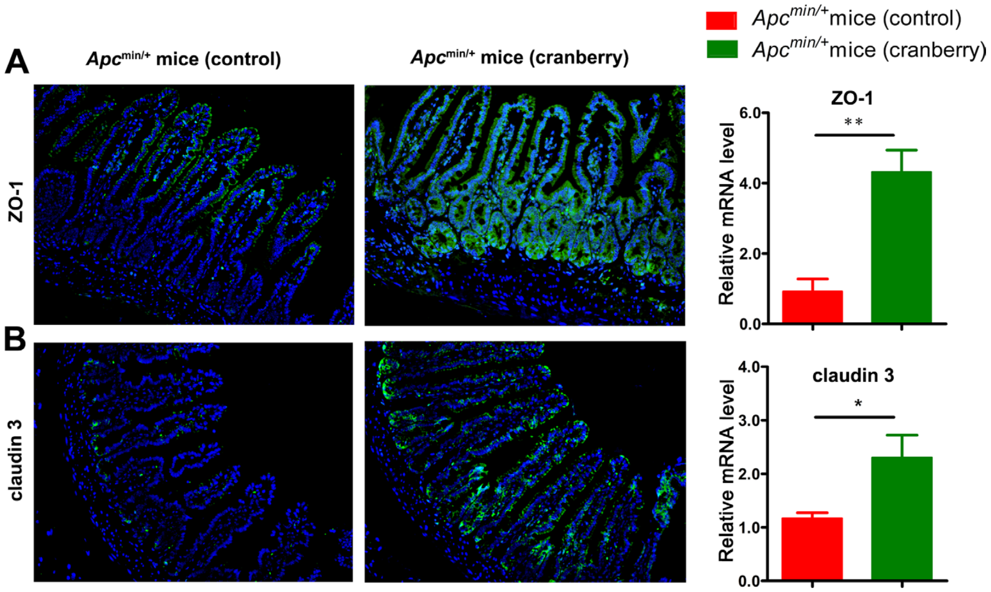

Supplementary Figure 1: Cranberry supplementation protected small intestinal barrier function. (**A**) Paraffin-embedded tissues from the middle small intestine were used to determine ZO-1 distribution by immunofluorescence stain using an anti-ZO-1 antibody and FITC-labeled secondary antibody and visualized using fluorescence microcopy (green staining; 200×). Nuclei were stained with DAPI (blue staining). Real-time PCR analysis of ZO-1 expression in the cell membranes of intestinal epithelial cells was shown. (**B**) Claudin 3 distribution was showed analogously by immunofluorescence stain and real-time PCR.$^{**}p < 0.01$, $^{*}p < 0.05$, cranberry diet-fed *vs* basal diet-fed *Apc*$^{min/+}$ mice. $n = 10$/group.

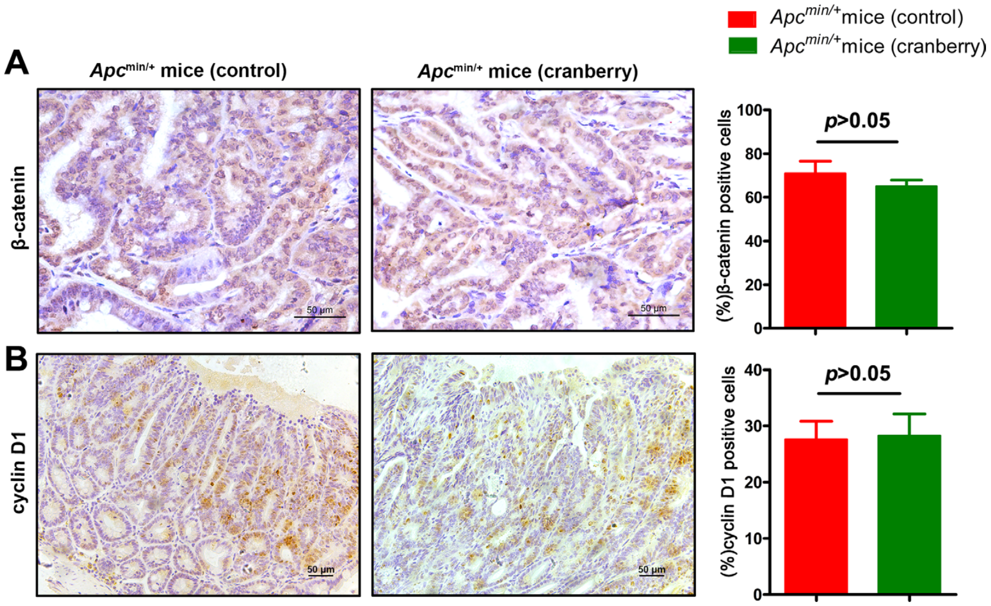

Supplementary Figure 2: Cranberry supplementation did not inhibit the activation of Wnt/β-catenin signaling in intestinal tumors. (**A**–**B**) β-catenin and cyclin D1from the middle small intestine of both groups were shown by immunohistochemical staining (400×; 200×). The positive staining of β-catenin located at cytoplasm and/or nuclear was included to quantify positive cells. Data were quantified as mean percentage of positive cells at five randomly selected fields in each section. Columns, means from at least six mice in each group; bars, standard deviation. Cranberry diet-fed *vs* basal diet-fed *Apc*$^{min/+}$ mice. Scale bars, 50 μm. $n = 8$/group.
